# Effects of endurance training on skeletal muscle mitochondrial respiration in Siberian huskies and Alaskan huskies

**DOI:** 10.14814/phy2.70725

**Published:** 2026-01-19

**Authors:** Silje Sælen‐Helgesson, Anne Dragøy Hafstad, Trine Lund, Ingebjørg Helena Nymo, Chiara Ciccone, Shona Hiedi Wood, Lars P. Folkow, Monica Alterskjær Sundset

**Affiliations:** ^1^ Department of Arctic and Marine Biology UiT The Arctic University of Norway Tromsø Norway; ^2^ Cardiovascular Research Group, Department of Medical Biology, Faculty of Health Science UiT The Arctic University of Norway Tromsø Norway; ^3^ Section of Food Safety and Animal Health Research Norwegian Veterinary Institute Tromsø Norway

**Keywords:** animal welfare, *Canis lupus familiaris*, citrate synthase activity assay, endurance training, high‐resolution respirometry, mitochondrial respiration

## Abstract

Siberian huskies (SH) and Alaskan huskies (AH), sharing ancestry with ancient sled dogs, were hypothesized to achieve similar skeletal muscle (SM) mitochondrial respiration capacities and densities through endurance training. High‐resolution respirometry of SM biopsies from SH and AH during off‐season (5 SH, 4 AH) and racing‐season (5 SH, 7 AH) revealed a striking increase in mass‐specific succinate‐linked mitochondrial complex II (CII) activity during racing‐season, in both SH (+75%) and AH (+129%). These increases were accompanied by increased protein content in SM for both SH (+37%) and AH (+56%). Elevated CII respiratory capacity can potentially reflect increased fatty acid utilization. NADH‐linked complex I (CI) respiration increased significantly only in AH (+35%), which also, unlike SH, exhibited significantly elevated citrate synthase activity (+270%). Both groups showed reduced protein‐specific residual oxygen consumption during racing‐season (SH: −45%, AH: −48%) and increased reactive oxygen species production. Together, these changes point to more efficient mitochondria with minimized energy loss in raced dogs. A minimally invasive sampling approach was validated, using NSAIDs, local anesthesia, light oral sedation, a micro biopsy gun, and individualized environments to minimize distress. This secured good animal welfare and provided a practical method for field‐based or repeated SM biopsies without general anesthesia.

## Graphical abstract

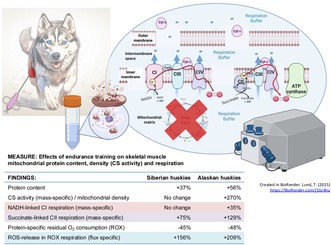

## 1 INTRODUCTION

Siberian huskies trace their lineage to ancient Arctic sled dogs from northeast Siberia, dating back 11,700 years (Feuerborn et al., 2021; Sinding et al., 2020; Smith et al., 2024). Genomic evidence identifies two distinct Arctic dog lineages emerging during transition from the glacial to interglacial period, shaped by human use for long‐distance transportation and hunting (Smith et al., 2024). Since their recognition as a breed by the American Kennel Club in 1930, a century of breeding for various purposes–racing lines (function), show lines (form), companion dogs (pets), and “dual purpose” pet‐sled and show‐sled dogs has significantly influenced the genetic structure of the Siberian husky population (Huson et al., 2025; Smith et al., 2024).

Alaskan huskies, though not a registered breed, also share substantial ancestry with the ancient Arctic sled dogs and are bred exclusively for endurance and performance (Huson et al., 2010; Smith et al., 2024; Thorsrud & Huson, 2021). They have dominated middle‐ and long‐distance races such as the Iditarod (~1600 km) in Alaska and Finnmarksløpet (~1200 km) in Norway. Teams of 12–16 dogs pull sleds with mushers, equipment, and food over vast distances under extreme Arctic conditions.

While Siberian husky teams also compete in these endurance events, they are underrepresented compared to Alaskan huskies. In 2025, Siberian husky teams nevertheless made history in the International Federation of Sleddog Sports World Championship Femundløpet in Norway by winning double gold and double silver, in the 450 km race held in conjunction with the Registered Nordic Breed (RNB) category (Sports IFoS, 2024), in which six of the top ten teams were Siberian husky teams. This success highlights the impact of the selective breeding of Siberian huskies for endurance, speed, and work ethics. A recent study also shows that genes involved in muscle organ development, lipid metabolism and glucose transport, lung vasculature development, limbs and bones development, eye structure, and pigmentation are enriched for in Siberian huskies from racing lines (Huson et al., 2025).

Sustaining such performances requires exceptionally high energy fluxes to support prolonged intense skeletal muscle (SM) activity. Mitochondria, responsible for ATP (adenosine triphosphate) production in SM, consist of ~1200 proteins primarily encoded by the nuclear genome (Oliveira & Hood, 2019) and exhibit remarkable plasticity in volume, structure, and function in response to exercise or lack thereof (Hood et al., 2019). The mitochondrial oxidative phosphorylation (OXPHOS) system, located in the inner mitochondrial membrane, produces ATP through the orchestrated actions of the electron transport system (ETS) including ATP synthase (Complex V (CV)) and two electron carriers (ubiquinone (coenzyme Q junction, CoO) and cytochrome c) (Gnaiger, 2009; Xu et al., 2020). Electron transfer via this system, in which Complex I (CI) and Complex II (CII, also called succinate dehydrogenase) play decisive roles, drives proton pumping across the membrane, creating an electrochemical gradient used by CV to generate ATP for muscle work and cellular activities. The tricarboxylic acid (TCA) cycle in the mitochondrial matrix produces reduced electron carriers, nicotinamide adenine dinucleotide (NADH) (for CI) and FADH_2_, from Acetyl‐CoA derived from glucose and other metabolites via glycolysis in the cytosol and from fatty acids via β‐oxidation (Gnaiger, 2024). Under normal aerobic conditions, the primary source of succinate is α‐ketoglutarate converted through the TCA cycle in the mitochondrial matrix (Chinopoulos, 2019). During the oxidation of succinate, CII reduces FAD to FADH_2_ (Gnaiger, 2024).

Acute exercise typically induces mitochondrial biogenesis (Huertas et al., 2019), with even single bouts altering mitochondrial morphology (Picard et al., 2013). High basal mitochondrial protein synthesis rates enable rapid reconfiguration of mitochondrial proteins and function in response to energetic challenges (e.g., intense exercise) and environmental extremes. Accordingly, using high‐respirometry and spanning the entire training and racing seasons, a study of Alaskan huskies clearly demonstrated an increase in mitochondrial functional capacity in raced dogs (Miller et al., 2017), indicating significant mitochondrial re‐modeling and increases in mitochondrial density. Thus, OXPHOS‐to‐ETS capacity ratio (P/E) was shown to increase, from 0.90 in non‐raced dogs to 0.97 after the 1600‐km Iditarod, indicating near‐complete matching of phosphorylation and ETS capacities (Miller et al., 2017). While human skeletal muscle (SM) OXPHOS capacities range from 60 to 180 pmol O_2_·s^−1^·mg wet tissue^−1^ depending on fitness levels (Gnaiger, 2009), the raced Alaskan huskies exhibited the highest OXPHOS (254 ± 26 pmol O_2_·s^−1^·mg wet tissue^−1^) and ETS capacities (254 ± 37 pmol O_2_·s^−1^·mg wet tissue^−1^) hitherto recorded in mammalian SMs (Miller et al., 2017).

High‐resolution respirometry studies of mitochondrial respiration in canine SMs have so far only been conducted in Alaskan huskies, using fresh SM biopsies obtained under general anesthesia (Davis & Barrett, 2021; Miller et al., 2017). Although this approach was previously limited to fresh samples (since freezing and thawing tissue disrupts and permeabilizes the inner mitochondrial membrane, leading to inactivation of the TCA cycle and release of electron carriers of the electron transport chain (Araki, 1977)), the use of Seahorse XF Analyser and high resolution respirometry methods to reliably assess mitochondrial oxygen consumption in previously frozen tissue has now been described (Acin‐Perez et al., 2020; Ebanks et al., 2023). In such protocols, cytochrome C is added to ensure electron transfer between the different complexes, and the specific substrate and inhibitor combinations allow for reliable measurements of respiration of specific complexes of the ETS (Ebanks et al., 2023; Hansen et al., 2022).

Here, we have used the Oroboros Oxygraph‐O2k to perform high‐resolution respirometry, using substrate‐uncoupler‐inhibitor titration (SUIT) protocols (Doerrier et al., 2018) to determine mitochondrial respiratory function in previously frozen SM samples from both Siberian and Alaskan huskies, either obtained off‐season or after endurance training and racing. We hypothesized that in sharing a common ancestry with ancient Arctic sled dogs and by being shaped by a long history of selection and breeding, both Siberian and Alaskan huskies can achieve equally high SM mitochondrial respiration capacities and mitochondrial densities in response to endurance training. An additional important objective was to evaluate a refined method for obtaining canine SM micro‐biopsies without use of general anesthesia (known to potentially cause adverse effects in Siberian huskies (Hawley & Wetmore, 2010)), thus promoting good animal welfare and allowing repeated and field‐based biopsy sampling (Hayot et al., 2005; Newmire & Willoughby, 2022).

The specific objectives were:

1. To compare the maximal activity of mitochondria respiratory CI and CII in previously frozen SM homogenates between Siberian and Alaskan huskies, and between racing‐season and off–season.
2. To assess the effect of endurance training on mitochondrial density in Siberian and Alaskan huskies, using measurements of citrate synthase (CS) activity as a proxy; and
3. To evaluate a refined approach for sampling muscle biopsies in dogs, employing a combination of a systemic non‐steroidal anti‐inflammatory drug (NSAID), light oral sedation, local anesthesia, a micro biopsy gun and individualized environments to minimize distress.

## 2 METHODS

### 2.1 Animals

Six Siberian huskies (racing lines) and seven Alaskan huskies from two collaborating kennels (Snykovet Siberian Husky Kennel and Mjaatvedt Husky) located outside Tromsø (69°39′ N 18°57′ E) in northern Norway were included in the study (Table 1). Eight of the dogs (SH1–4 and AH1–4) were trained and raced together as one team during 2021/2022, completing Bergebyløpet (240 km, average speed 11.9 km·h^−1^) in February 2022 and Finnmarksløpet (600 km, average speed 9.5 km·h^−1^) in March 2022. In the following year (2022/2023), the Siberian huskies (SH1–6) and the Alaskan huskies (AH1–7) were trained and raced separately as individual teams (Table 1). The animals were sampled in August 2022 (off‐season) and March 2023 (racing‐season).

**TABLE 1:** Animals included in the study, all of which were intact and not neutered.

Animal ID	Sex	Birth year	Sampled off‐season (body mass) (August 14–18 in 2022)	Sampled during the racing‐season (body mass) (March 16–25 in 2023)****
Siberian huskies				
SH1 (Nova)	F	2019	No (used in breeding)	Yes (21 kg)
SH2 (Skádja)	F	2015	Yes (21.5 kg)	Yes (20 kg)
SH3 (Chloe)*	F	2016	Yes (19 kg)	Yes (18 kg)
SH4 (Lala)*	F	2016	Yes (18.5 kg)	Yes (18.5 kg)
SH5 (Albbas)**	M	2021	Yes (22 kg)	No (re‐homed)
SH6 (Balder)**	M	2021	Yes (22.3 kg)	Yes (20 kg)
Alaskan huskies				
AH1 (Lakris)***	M	2019	Yes (26 kg)	Yes
AH2 (Rafael)***	M	2019	Yes (22 kg)	Yes
AH3 (Idun)	F	2012	Yes (23 kg)	Yes
AH4 (Luna)***	F	2019	Yes (23 kg)	Yes
AH5 (Zelda)***	F	2019	No	Yes (18 kg)
AH6 (Mario)	M	2016	No	Yes (22 kg)
AH7 (Hydro)	M	2016	No	Yes (20 kg)

Abbreviations: F, females; M, males.

* Sisters from the same litter.

** Brothers from the same litter, cousins to SH1.

*** Siblings after AH3.

**** The Siberian husky team completed four races prior to sampling: Beaskádas (245 km, average speed 11.4 km·h^−1^), and TromsQuest (60 km, 15 km·h^−1^km/h) in January, Bergebyløpet (240 km, 10.6 km/h) in February, and Herringen Trail (50 km, 12.3 km·h^−1^) in March. The Alaskan husky team completed one race prior to sampling: TromsQuest (180 km, 13.8·km·h^−1^) in January.

The dogs were kept in outdoor facilities year‐round. Two to three dogs shared secure yards (~15 m^2^) with wooden floors or gravel, shadow from the sun, place to rest and play, and dry, wooden houses with wood wool or dried hay (~80 × 60 × 70 cm). During the training season two to three meals were provided per day (meat soup consisting of dry protein kibbles together with raw meat mixed with water). Kibbles used for the Siberian huskies contained 35% protein, 23.5% carbohydrates, and 21% fat (Virbac veterinary HPM baby, Virbac, France). Kibbles used for the Alaskan huskies contained 28% protein, 28% carbohydrates, 21% fat (Sporting life energy 4300, Royal Canin, France). In addition, they received raw meat consisting of 15% protein and 10% fat (VOM Active, VOM og Hundemat, Norway), vitamin B, omega 3 oil, and digestive support powder. During the summer (off‐season) the dogs were fed two times a day. In addition to regular feedings, they also received treats and chewing stimuli such as dried fish and whole meat year‐round.

Training intensities were gradually reduced in spring from April to May, leading up to the off‐season. During warmer days activities were restricted to the afternoon when temperatures had dropped. Summer activities included swimming, hiking, biking, cuddles, and playing. Autumn training started when temperatures were sufficiently low (usually below 12°C). Training distance, duration, and intensity increased during the autumn to prepare the dogs for the sled dog races in January to April. Training was conducted with a cart during autumn and a sled when snow conditions allowed for this and was logged by the mushers to keep track of the progression and recovery needed. From July to February 2021/2022 the joint team of Alaskan and Siberian huskies completed 2700 km of training in total on dry land and snow. From August to March in 2022/2023 the Siberian Husky team completed 3700 km of training on dry land and snow, prior to sampling in March, while the Alaskan husky team completed 2500 km during the same time frame. A canine physiotherapist checked the dogs when needed, in addition to veterinary checkups and vaccinations. Massages were also used after training to ensure proper muscle recovery.

### 2.2 Muscle biopsies and animal welfare considerations

SM biopsies (approximately 50 mg) were taken from *musculus biceps femoris*, a large, superficial muscle extending from the hip joint in the hindlimb (Carpenter & Cooper, 2000; Evans & Lahunta, 2013). This muscle is highly endurant, involved in locomotor activity in long‐distance sled dogs, and therefore of particular interest in terms of mitochondrial respiration (Miller et al., 2017). Biopsies were collected off‐season when training activity was lower (August 2022), from SH2–6 (SH1 was excluded due to a planned litter of puppies born August 19, 2022) and AH1–4 (Table 1). During the racing season, five Siberian huskies (SH1–4 and SH6) were included in the sampling (SH5 was excluded due to rehoming) (Table 1). The initial data from the August sampling showed large individual variations in mitochondrial respiration among the Alaskan huskies. Three additional dogs (AH5–7) were consequently included in the second sampling, in March 2023, to secure more statistical power. Hence, a total of seven Alaskan huskies were sampled during the racing season (Table 1).

**FIGURE 1:**
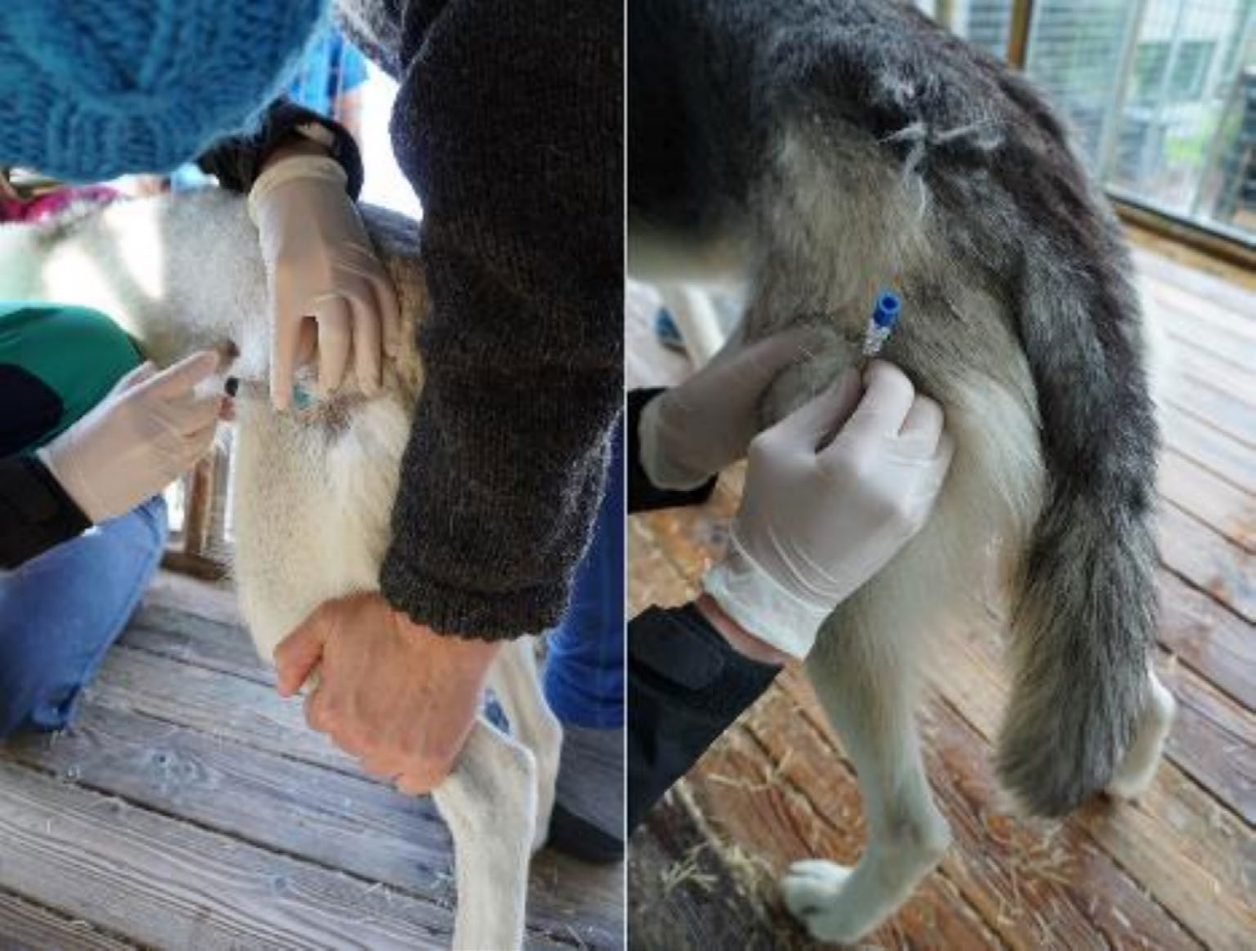
Injection of the local anesthetics to the left, and the core biopsy needle to the right.

NSAID firocoxib (Previcox 227 mg, 5 mg·kg^−1^ PO, Boehringer Ingelheim Vetmedica GmbH, Cat. No: QM0aA H90) was administered 2 hours before the biopsy procedure and subsequently every 24th hour for the next 2 days. The sedative dexmedetomidine hydrochloride oromucosal gel (Sileo® mouth gel 0.1 mg·mL^−1^, 125 μg·m^−2^, Orion Corporation, Cat. No: QNO5CM18) (Korpivaara et al., 2021) was given on the oral mucosa 30 minutes prior to the biopsy procedure. The dogs stood during the biopsy procedure. They were given treats and cuddles while gently held in place by two people from the kennel, including the musher. A small area (approximately 2.5 × 2.5 cm) of the skin covering the proximal part of the *m. biceps femoris* was shaved, washed with chlorhexidine (Hibiscrub, 40 mg·mL^−1^; Mölnlycke Health Care AB, Cat. No: 596023) or cetylpyridin (Pyrisept, 1 mg·mL^−1^; Karo Pharma, Cat. No: D08A J03), disinfected with 70% ethanol using sterile compresses and allowed to dry. The skin and overlying fascia were then infiltrated with 4–6 mL lidokainhydroklorid/adrenalin (Xylocain‐Adrenalin 10 mg·mL^−1^ + 5 μg·mL^−1^) in an L‐ Block (1.5 mL subcutaneously in each direction, and 1 mL in the middle of the L‐shape, penetrating a little deeper) (Figure 1).

The skin was disinfected again with 70% ethanol prior to the sampling, and a spring‐loaded one‐handed automated system from Merit Medical Systems, USA (CTT1411 TEMNO Evolution Coaxial Bundle 14 G. 11.0 cm, Coax Bundle 16.0 cm 13.5 G) was used for the micro biopsy (Gerth et al., 2009).

**FIGURE 2:**
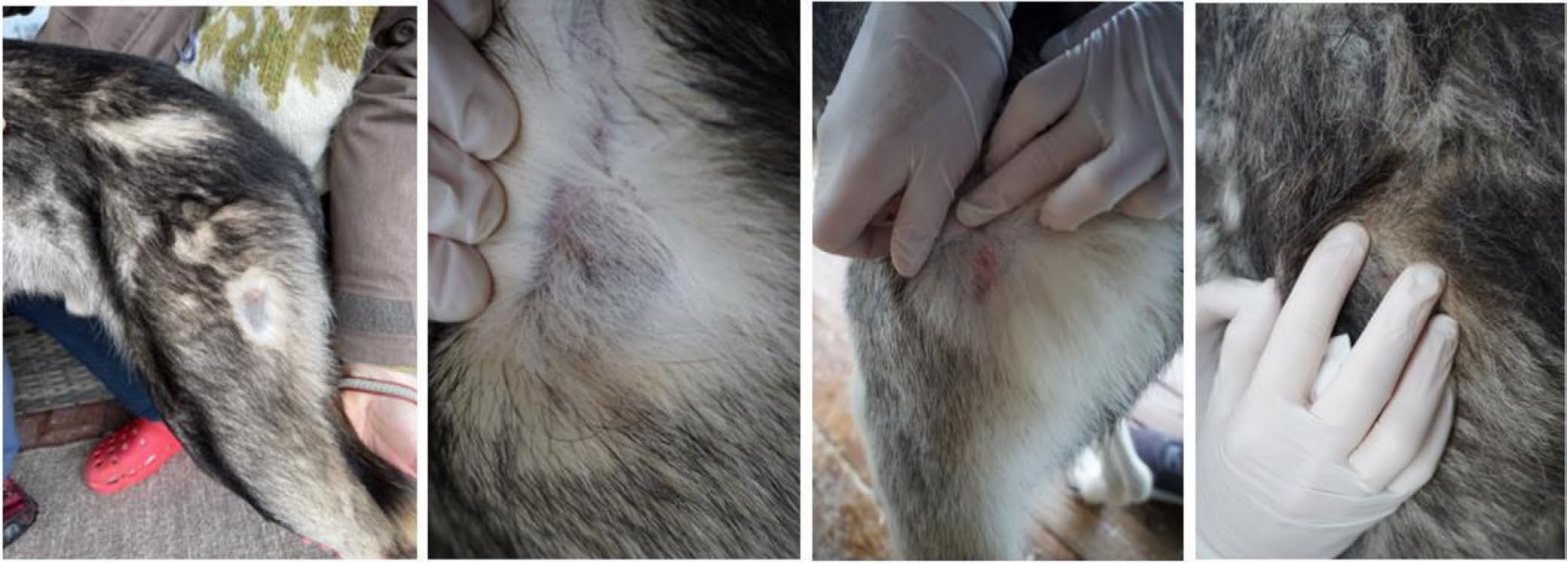
Post‐biopsy wound. Pictures taken directly after the core biopsy needle was removed, and the puncture site cleaned.

The introducer needle was inserted by the veterinarian into the proximal *m. biceps femoris* with a penetration depth of 1 cm. The introducer needle was then removed, leaving a core biopsy needle in place (Figure 1). The trigger needle was then introduced through the core needle and pre‐set to obtain approximately 20 mg of tissue. Unloaded with a spring, the needle fired into the muscle, obtaining a small piece of tissue. The trigger needle was introduced twice for most dogs, collecting 40–50 mg of SM tissue in total. For two dogs, the needle was introduced three times to yield enough SM tissue. The core needle was then removed, and the puncture site examined, cleaned, and documented with photographs (Figure 2). The puncture site was thereafter examined daily for 2 days. The muscle tissue was immediately transferred to a tube containing ice cold BIOPS solution (10 mM Ca‐EGTA buffer, 0.1 μM free calcium, 20 mM imidazole, 20 mM taurine, 50 mM K‐MES, 0.5 mM DTT, 6.56 mM MgCl_2_, 5.77 mM ATP, 15 mM phosphocreatine) and put on ice.

### 2.3 Muscle homogenate

A protocol for respiratory measurements in fresh SM homogenate was initially followed (Draxl et al., 2013), but due to experimental challenges, the homogenates were frozen for later analysis. Briefly, SM samples were wiped removing excess fluid, weighed, and adjusted to 50 mg of tissue, before being placed in a new container with 500 μL of fresh ice‐cold BIOPS. All further steps were conducted on ice. The tissue was cut into small pieces, before adding trypsin (Cat. No: 15090046, Thermo Fisher Scientific, Massachusetts, USA) at a final concentration of 0.25% and incubating for 20 min. The liquid containing BIOPS and trypsin was carefully removed and the sample washed two times with BIOPS without removing the tissue from the container.

The tissue was placed on the small disc of a shredder tube. The small chamber was then closed with a tube tool, turned upside down, and the big chamber filled with 500 μL of premade respiratory medium MiR05 (0.5 mM EGTA, 3 mM MgCl_2_‐6 H_2_O, 60 mM Lactobionic acid, 20 mM Taurine, 10 mM KH_2_PO_4_, 20 mM HEPES, 110 mM D‐Sucrose, 1 g·L^−1^ BSA) to preserve mitochondrial function. The amount of MiR05 was adjusted to the amount of tissue to form a 10% homogenate. The shredder tube was placed on the shredding base in position 1 and stirred for 10–12 s. After that, the sample was collected with a pipette and transferred to an Eppendorf tube and centrifuged (Himac CT15RE centrifuge, Hitachi Koki Co., Ltd) for 1 min at 268 relative centrifugal force (*g*) at 4°C. The supernatant was transferred to a new Eppendorf tube and stored at −70°C.

### 2.4 Mitochondrial respiration in pre‐frozen samples

The frozen SM homogenate (−70°C) was thawed and kept on ice until analyzed in the O2k FluoRespirometer (0.5 mL chambers, Oroboros Instruments, Austria). Respiration buffer MiR05 supplemented with 0.1 mM cytochrome C (Cat. No: C7752, Sigma Aldrich) and 20 U·mL^−1^ Superoxide dismutase (SOD, Cat. No: S8409, Sigma Aldrich) was added to chambers, followed by a 30 min equilibration of the respiration buffer with the partial pressure of the oxygen in the surroundings (air calibration).

The tissue homogenate (10 μL) was injected into the chambers with a Hamilton syringe (Hamilton Company, Nevada, USA), after approximately 5 min stabilization at 37°C with a stirred speed at 750 rotations per minute. Oxygen concentrations (mean ± SD) across experimental groups were 186.0 ± 5.54 μM at the start and 138.9 + 23.7 μM at the end of the analysis (Table 2). Separate protocols to measure CI (P_CI_) and CII (P_CII+ROS_) respiration were performed. To study CI activity, NADH (Cat. No: N8129, Sigma Aldrich) was used as an electron donor at a final concentration of 2.9 mM to feed electrons directly to CI, and oxygen flux (pmolO_2_·s^−1^·mL ^−1^) was recorded after stabilization of the O_2_ flux value.

**TABLE 2:** Chamber oxygen concentrations during the respirometry analysis from Protocol CI (P_CI_) using NADH as substrate and Protocol CII (P_CII_) using succinate as substrate.

Siberian husky, animal ID	μM O_2_ start	μM O_2_ end	Alaskan husky animal ID	μM O_2_ start	μM O_2_ end
Racing‐season_P_CI_			Racing‐season_P_CI_		
SH1	181.9	114.9	AH2	187.0	129.1
SH2	183.7	74.6	AH3	181.9	138.8
SH3	194.9	150.2	AH4	182.2	112.7
SH4	178.7	125.1	AH5	184.1	81.3
SH6	187.8	118.5	AH6	187.6	118.8
			AH7	174.2	138.6
Mean ± SD	185.4 ± 6.25	116.7 ± 27.26		182.8 ± 4.85	119.9 ± 21.61
Racing‐season P_CII±ROS_			Racing‐season P_CII+ROS_		
SH1	184.4	137.2	AH2	187.9	131.3
SH2	182.6	104.9	AH3	181.5	158.9
SH3	198.3	168.7	AH4	185.5	142.9
SH4	184.2	141.1	AH5	184.1	109.6
SH6	184.4	137.7	AH6	187.1	136.3
			AH7	185.9	152.4
Mean ± SD	186.8 ± 6.49	116.7 ± 27.26		182.8 ± 4.85	138.6 ± 17.44
Off‐season_ P_CI_			Off‐season_ P_CI_		
SH2	186.2	139.2	AH1	177.8	139.7
SH3	176.0	137.8	AH2	181.9	142.1
SH4	191.9	157.2	AH3	184.9	149.0
SH5	191.3	139.4	AH4	183.7	132.8
SH6	189.4	128.4			
Mean ± SD	187.0 ± 6.52	140.4 ± 10.43		182.1 ± 3.11	140.9 ± 6.69
Off‐season P_CII±ROS_			Off‐season P_CII±ROS_		
SH2	186.7	163.3	AH1	188.2	166.4
SH3	184.0	161.0	AH2	190.2	167.7
SH4	202.3	186.4	AH3	186.7	168.2
SH5	196.0	174.6	AH4	185.7	160.9
SH6	187.4	157.1			
Mean ± SD	191.3 ± 7.63	168.5 ± 11.95		187.7 ± 1.96	165.8 ± 3.35

The production of H_2_O_2_ was used as a proxy of the production of reactive oxygen species (ROS) in SM homogenates and was measured in the same protocol as CII‐linked respiration, by adding 10 μM Amplex Ultra Red (Cat. No: A36006, Invitrogen) and 1 U·mL^−1^ horseradish peroxidase (Cat. No: P8250, Sigma Adrich) (Makrecka‐Kuka et al., 2015). A H_2_O_2_ standard curve was generated by adding a commercial H_2_O_2_ solution (Cat. No: H1009, Sigma Aldrich) in three 0.16 μM steps. Then 10 μL homogenate was added, followed by rotenone (0.5 μM, Cat. No: R8875, Sigma Aldrich) to inhibit CI activity, and then addition of 10 mM succinate (Cat. No: S2378, Sigma Aldrich) as an electron donor to CII. Antimycin A (2.5 μM, Cat. No: A8674, Sigma Aldrich) was added to inhibit ETC. The protocol was ended by adding 10 μM S3Q EL2 (Cat. No: SML1554 Sigma Adrich), which inhibits ROS production from CIII.

### 2.5 Mitochondrial citrate synthase activity in pre‐frozen samples

CS activity is a quantitative biomarker used to determine mitochondrial density in SMs (Vigelsø et al., 2014), often measured using the tissue homogenate initially prepared for the Oroboros to track training‐induced changes in mitochondrial density (Eigentler et al., 2015; Wiegand & Remington, 1986). The stored homogenate at −70°C in MiR05 was analyzed for CS activity according to Hafstad et al. (2011). Briefly, a 4 μL aliquot of the homogenate sample was added to an assay buffer with 0.25% Triton X‐100, 0.5 mM oxaloacetate dissolved in a 0.1 M triethanolamine‐HCl buffer (pH 8.0) and 0.31 mM acetyl coenzyme A. The enzyme reaction was initiated by adding 0.1 mM 5,5′‐Dithiobis (2‐nitrobenzoic acid) (DTNB, Sigma‐Aldrich Cat. No: D8130), which was dissolved in 1 M Tris–HCl at pH 8.1. The CS activity was determined by measuring optical density (OD) at 412 nm every 15 seconds over a period of 2 min using a microplate reader (BioTek 800TS). The CS activity is reported as nmol·mL^−1^·min^−1^. All chemicals used in the assay were purchased from Sigma Aldrich.

### 2.6 Analyzing and normalizing the respiration data

The data was analyzed using DatLab software (version 7.4.04) and normalized to homogenate wet weight (mass‐specific), citrate synthase activity (CS‐specific), or protein concentration (protein‐specific) measured by Bradford Assay (Bio Rad, California, USA). Graph prism was used for statistical analyses and figures. Between‐groups comparisons were made using a mixed model two‐way analysis of variance (ANOVA). The Shapiro–Wilk test was used to check normality of residuals. Data are expressed as means ± SD, and *p* ≤ 0.05 was taken to reflect a statistically significant difference.

## 3 RESULTS

### 3.1 Animal welfare considerations

Dexmedetomidine hydrochloride oromucosal gel is supposed to have a calming effect on dogs and was given 30 min prior to the micro biopsy procedure but did not have a notable effect on the behaviour of the dogs in this study. Still, none of the dogs showed any sign of distress or pain during the insertion of the biopsy needle or the biopsy sampling. Hence, the combination of the NSAID firocoxib and local anaesthesia with lidocaine hydrochloride/adrenalin appeared efficient in ensuring appropriate pain management. We observed that some of the dogs were more at ease and relaxed when sampled in their enclosures surrounded by their own pack, while others were happy to do the sampling indoors in the house of the owner. We adjusted the location for sampling accordingly, based on our observations and knowledge about the personality of individual dogs. We observed a haemorrhage in the insertion tube on several dogs during the biopsy procedure in March 2023, which we considered to result from a larger muscle mass and vascularization due to training, but no major haemorrhage was detected after removal of the needle. Only a minor haemorrhage was observed in two dogs after removal of the insertion tube, which stopped after a brief period (a few min) of digital press. Post‐sampling, there were no signs of infection, limping or general discomfort, and the small injection wounds healed quickly within the first few days.

### 3.2 Protein content and citrate synthase activity

**FIGURE 3:**
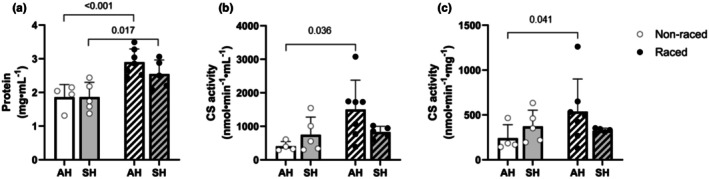
Protein concentration (a) and citrate synthase (CS) activity in 10% SM homogenate (b), and CS activity related to protein content (c), in skeletal muscle biopsies from Alaskan huskies (AH) and Siberian huskies (SH) during the off‐season period (non‐raced) and during the racing season (raced). Data are presented as single values and mean ± SD. *p*‐values for significant differences between groups are indicated (two‐way ANOVA).

There was a significant increase from the off‐season to the racing‐season in protein content in SM homogenates from both Siberian huskies (+32% from 1.9 ± 0.2 to 2.5 ± 0.2 mg·mL^−1^) and Alaskan huskies (+53% from 1.9 ± 0.2 to 2.9 ± 0.2 mg·mL^−1^) (Figure 3a). To assess mitochondrial density in the SM biopsies, we performed CS activity assays in the homogenates and related data to both homogenate volume (as a proxy for wet weight) (Figure 3b) and protein content (Figure 3c). In terms of CS activity, only Alaskan husky samples showed a significant increase during the raced season, both when related to wet weight (Figure 3b, +270% from 405 ± 71 to 1500 ± 382 nmol·mL^−1^·min^−1^) and to protein content (Figure 3c, +124% from 240 ± 76 nmol·mg^−1^·min^−1^ to 537 ± 161 nmol·mg^−1^·min^−1^). There were no significant differences in protein content or SM CS activity between Siberian and Alaskan huskies, neither in the racing‐season nor off‐season.

**FIGURE 4:**
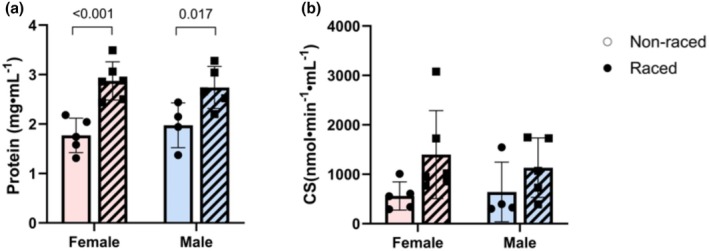
Protein concentration (a) and citrate synthase (CS) activity in 10% SM homogenates (b) obtained from skeletal muscle biopsies from female and male Alaskan and Siberian huskies combined, during the off‐season period (non‐raced) and during the racing season (raced). Data are presented as single values and mean ± SD. *p*‐values for significant differences between groups are indicated (two‐way ANOVA).

The racing teams studied herein consisted of both males and females – as is often the case. Sex was not a prespecified factor in this exploratory study, and we could not test for significant differences between males and females within each dog type due to the limited number of animals. But when pooling all dogs, no differences were found in SM protein content between males and females, neither off‐season (1.8 ± 0.2 mg·mL^−1^ for females and 2.0 ± 0.2 mg·mL^−1^ for males) nor during the racing season (2.9 + 0.2 mg·mL^−1^ for females and 2.7 + 0.2 mg·mL^−1^ for males, Figure 4a). Also, no differences were seen between males and females in terms of CS activity in SM homogenates, with a non‐significant trend for increased CS activity in the racing season in both sexes (Figure 4b).

### 3.3 Respiration rates in pre‐frozen SM homogenates

**FIGURE 5:**
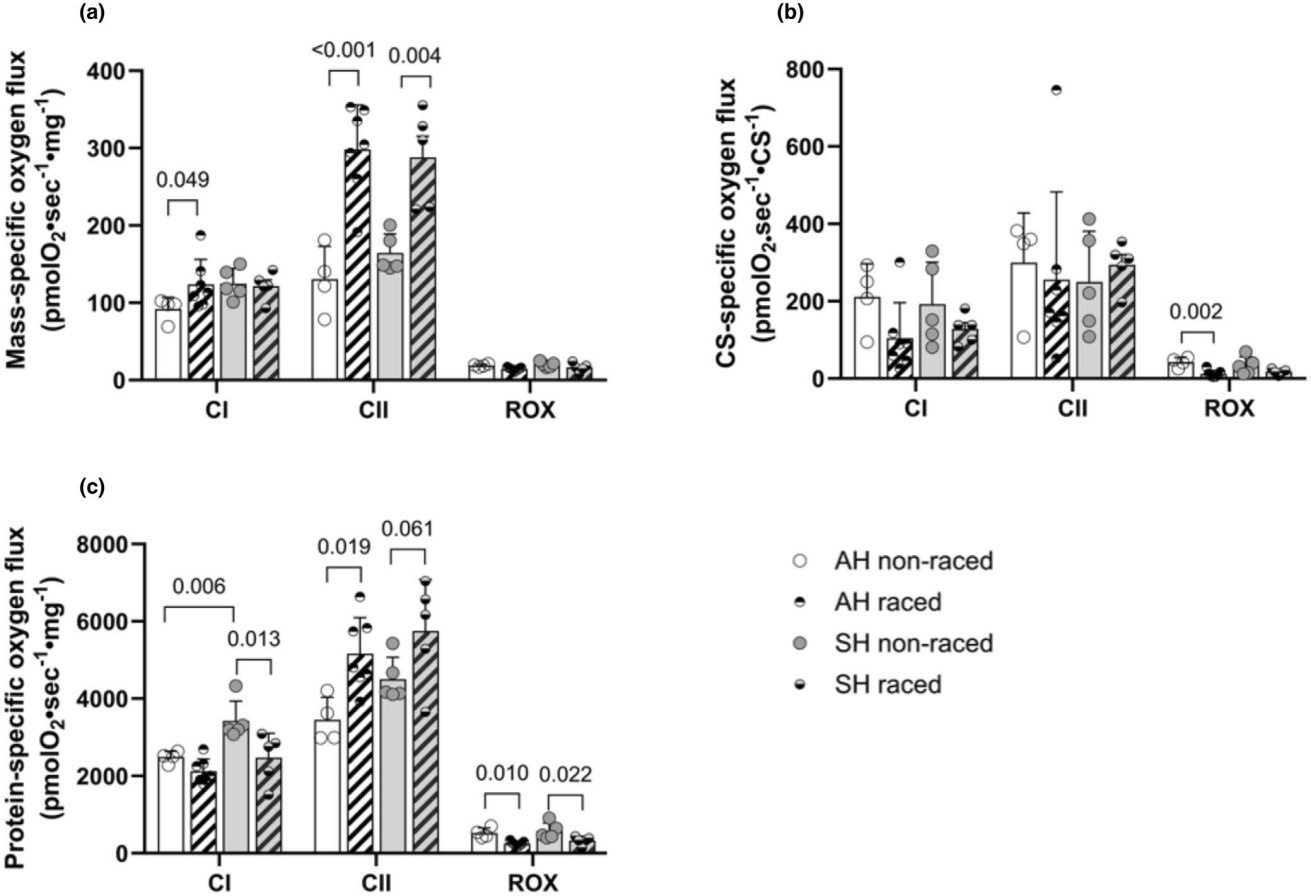
Respiration in pre‐frozen skeletal muscle homogenates. Mass‐specific (per mg wet weight, a), citrate synthase (CS, in pmol O_2_·s^−1^·mL^−1^)‐specific (b) and protein‐specific (c) respiration rates in SM homogenates from Alaskan huskies (AH) and Siberian huskies (SH) during off‐season period (non‐raced) and racing season (raced). Respiration was measured in homogenate with cytochrome C and CI substrate; NADH (CI), or in a separate protocol the CII substrate; succinate (CII), followed by addition of CI‐blocker rotenone and CIII‐blocker antimycin to obtain residual oxygen consumption (ROX). Data are presented as single values and mean ± SD. *p*‐values for significant differences between groups are indicated (two‐way ANOVA).

All respiratory data were obtained from pre‐frozen (at −70°C) SM homogenates and were analyzed together within a short timeframe (2 weeks). We have normalized oxygen fluxes in the homogenates, as measured in the O2k FluoRespirometer, to wet weight (mass‐specific, Figure 5a), to CS activity (CS‐specific, Figure 5b), and to protein content in the homogenates (protein‐specific, Figure 5c). No significant difference was found in the different respiratory states between Siberian and Alaskan huskies in the racing season (Figure 5). Siberian huskies did, however, exhibit higher protein‐specific CI respiration than did Alaskan huskies during the off season (Figure 5c).

A significant increase was seen in raced as compared to non‐raced individuals both in Siberian and Alaskan huskies in terms of mass‐specific (+75% and +129%, respectively, Figure 5a) and protein‐specific (+27% and +49%, respectively, Figure 5c) CII respiratory capacity (Figure 5a,c). There was, however, no difference in CS‐specific CI or CII‐respiration, neither between the two groups nor between seasons (Figure 5b).

We also found a smaller (+35%), but significant *increase* in mass‐specific CI respiration in Alaskan huskies, from 92 ± 8 pmol O_2_·s^−1^·mg wet tissue^−1^ in non‐raced to 124 ± 14 pmol O_2_·s^−1^·mg wet tissue^−1^ in raced dogs (Figure 5a), while CI respiration related to protein content did not differ between seasons (Figure 5c). This was not observed for raced Siberian huskies, in which protein‐specific CI respiration was found to be significantly *reduced* compared to off‐season (−28%), from a non‐raced SH value that was significantly higher than in non‐raced AH (Figure 5c).

Both Siberian and Alaskan huskies showed significant reductions in protein‐specific ROX respiration during the racing season (−62% and −58%, respectively, Figure 5c).

**FIGURE 6:**
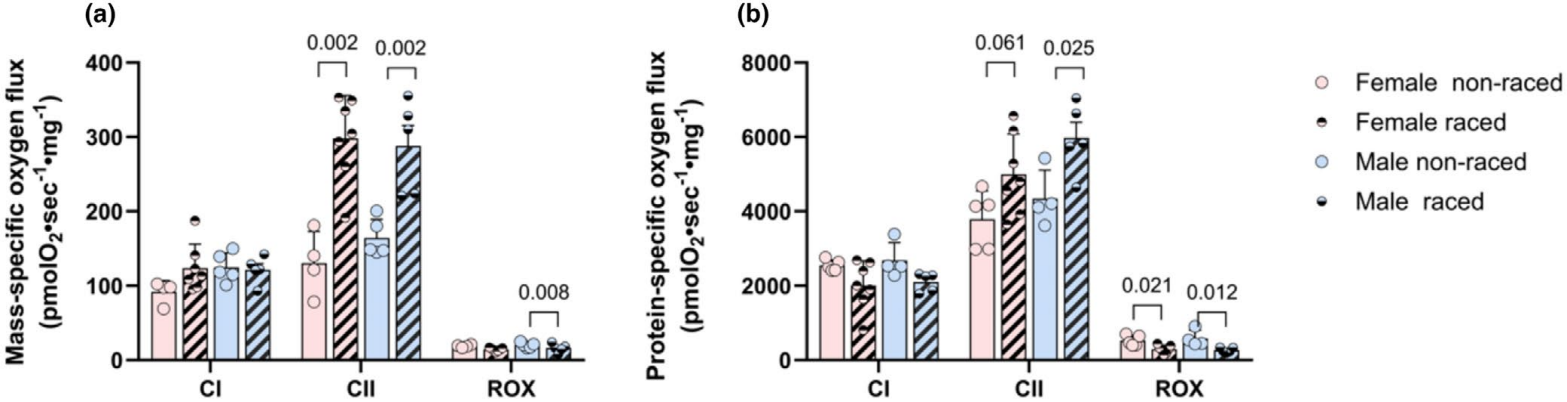
Respiration in pre‐frozen skeletal muscle homogenate. Mass‐specific (per mg wet weight, a), and protein‐specific (b) respiration rates in SM homogenates from female and male Alaskan‐ and Siberian huskies combined, during off‐season period (non‐raced) and racing‐season (raced). Respiration was measured in homogenate with cytochrome C and CI substrate; NADH (CI) or in a separate CII substrate protocol; succinate (CII) followed by addition of CI‐blocker rotenone and CIII‐blocker antimycin to obtain residual oxygen consumption (ROX). Data are presented as single values and mean SD. *p*‐values for significant differences between groups are indicated (two‐way ANOVA).

No difference was found in mass‐ and protein‐specific respiration (CI, CII, or ROX) between sexes in the pooled data, either in the off‐season or in the racing‐season, but there were similar significant differences in CII and ROX respiration between seasons as in the non‐pooled data for both sexes (Figure 6a,b).

**FIGURE 7:**
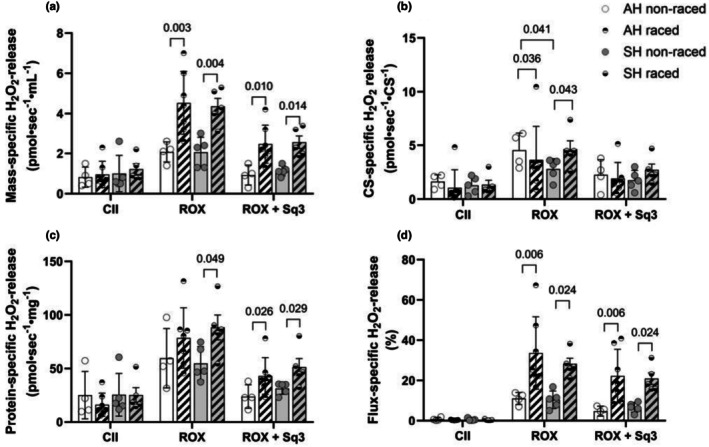
ROS‐release in pre‐frozen husky skeletal muscle homogenates. Mass‐specific (per mg wet weight, a), citrate synthase‐specific (CS) (b), protein‐specific (c), and flux‐specific (D) H_2_O_2_‐release in SM homogenates from Alaskan huskies (AH) and Siberian huskies (SH) during off‐season (non‐raced) and racing‐season (raced). H_2_O_2_ release was measured following the addition of cytochrome C and the CII substrate succinate (CII), followed by addition of CI‐blocker rotenone and CIII‐blocker antimycin to obtain residual oxygen consumption (ROX). ROS‐inhibitor sequel 3 (ROX + Sq3) was added to block ROS production from CIII. Data are presented as single values and mean ± SD. *p‐values for significant differences between groups are indicated (two‐way ANOVA).

There were no differences in ROS‐release (using H_2_O_2_‐production as a proxy) between groups when SM homogenate was respiring on succinate (CII‐substrate) (Figure 7). However, following the inhibition of CI and CIII, we observed a significant increase in mass‐ and flux‐specific H_2_O_2_‐production in the ROX states in raced vs. off‐season dogs, in both Siberian and Alaskan huskies (Figure 7a,d). The same trends were seen in protein‐specific ROS‐release, but only significant in the Siberian husky group (Figure 7c). Adding an inhibitor of CIII ROS production (Sequel 3) reduced overall ROS production, but the significant differences between animals during off‐season and the racing‐season remained. There were less clear differences in CS‐specific ROS‐release between animals during off‐season and racing‐season (Figure 7b). However, there was more CS‐specific ROS‐release in the Alaskan husky group than in the Siberian husky group in the off season in the ROX‐state. Curiously, CS‐specific ROS release in the ROX‐state was significantly reduced in the Alaskan husky group, while it increased in the Siberian husky group in the racing season as compared to the off‐season.

**FIGURE 8:**
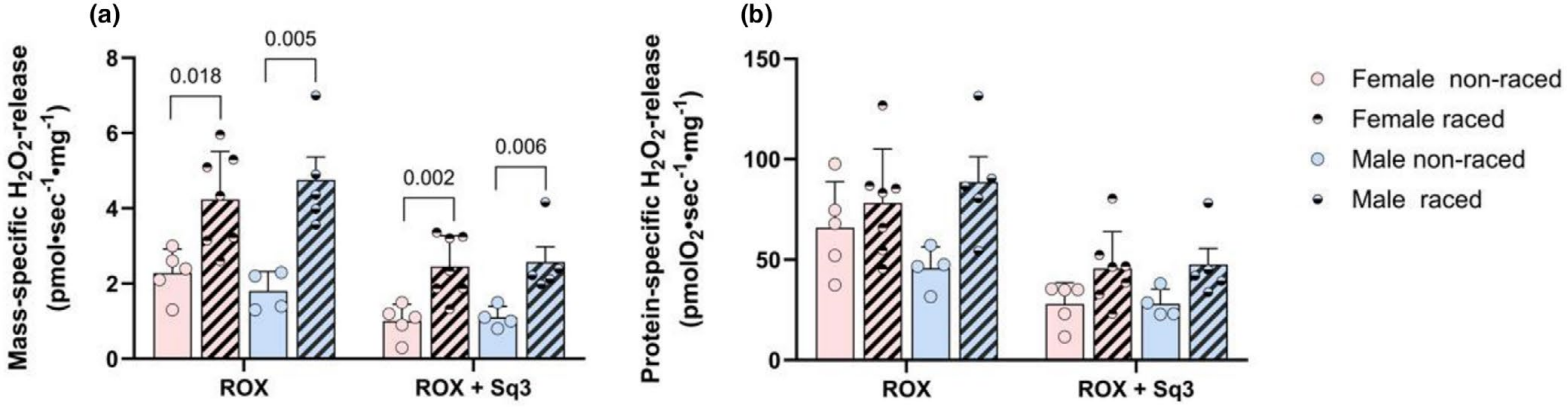
ROS‐release in pre‐frozen skeletal muscle homogenates. Mass‐specific (per mg wet weight, a) and protein‐specific (b) H_2_O_2_‐release in SM homogenates from female and male Siberian and Alaskan huskies combined during off‐season period (non‐raced) and racing season (raced). H_2_O_2_ release was measured following addition of cytochrome C and the CII substrate: succinate, followed by addition of CI‐blocker rotenone and CIII‐blocker antimycin to obtain residual oxygen consumption (ROX). ROS‐inhibitor sequel 3 (ROX + Sq3) was added to block ROS production from CIII. Data are presented as single values and mean ± SD. *p*‐values for significant differences between groups are indicated (two‐way ANOVA).

In line with the respiratory data, the trends for H_2_O_2_‐ release from respiring SM homogenate were similar between sexes in terms of mass‐ and protein‐specific ROS release (Figure 8a,b). Thus, both sexes showed a prominent increase in mass‐specific ROS production in the racing season (Figure 8a), with the same (but non‐significant) trends observed for protein‐specific ROS production (Figure 8b).

## 4 DISCUSSION

Different skeletal muscle (SM) mitochondrial phenotypes are present in early life, influenced by breeding selection (in the case of animals bred for racing) and later refined by exercise training and nutritional inputs impacting the performance of competitive athletes (Latham et al., 2022). Gaining knowledge of muscular mitochondrial biology can help inform breeding, training and nutrition to optimize performance and decrease incidence of fatigue‐induced injuries in canine and equine athletes. This exploratory study is the first to investigate the effect of endurance training on SM mitochondrial densities and respiration in Siberian huskies (SH) as compared to Alaskan huskies (AH). Both demonstrated a striking increase in succinate‐linked mitochondrial CII activity (SH: +75%, AH: +129%) and protein content, while NADH‐linked CI respiration increased only in Alaskan huskies (+35%). Alaskan huskies also exhibited elevated citrate synthase activity (+270%) indicative of increased mitochondrial density (Meinild Lundby et al., 2018; Vigelsø et al., 2014). Both Siberian and Alaskan huskies showed reduced protein‐specific residual oxygen consumption during the racing season, suggesting more efficient mitochondria with minimized energy loss. Together, these responses to endurance training suggest enhanced SM mitochondrial efficiency and fatty acid utilization, though they were accompanied by increased reactive oxygen species production, as discussed below. This study also reports the positive result of an alternative biopsy sampling method using local anesthesia in awake dogs.

### 4.1 SM protein content and CS activity

Exercise is known to increase protein content (Egan et al., 2013), mitochondrial gene expression (Egan et al., 2013; Petrella et al., 2008; Siu et al., 2003) and density (Gibala et al., 2006) in SMs in general, leading to improved resistance towards muscle fatigue. In humans, Meinild Lundby et al. (2018) documented a 44 ± 12% increase in CS activity in SM among 21 healthy males after 6 weeks of endurance training, while the OXPHOS capacity remained unchanged. The study also demonstrated that exercise training increased mitochondrial volume density (Mito_VD_) in SM, primarily through the enlargement of existing mitochondria, rather than de novo biogenesis. Furthermore, CS activity was strongly correlated with Mito_VD_, leading the authors to conclude that this assay is a reliable indicator for tracking training‐induced changes in Mito_VD_.

Miller et al. (2017) concluded that Alaskan huskies maintain a high mitochondrial protein turn‐over facilitating rapid muscle re‐modeling in response to environmental extremes and energetic challenges during racing. In the present study, a significant increase in the SM protein content was also observed, both in Siberian (+37%) and Alaskan huskies (+56%) during racing‐season, as compared to off‐season. However, only Alaskan huskies showed a large significant increase in SM CS activity during racing‐season vs. off‐season, both in total (+270%) and when related to protein content (+124%). Our dogs were sampled *during* the racing‐season‐but not straight after a race or a training session. The SM CS activities reported herein consequently do not reflect acute changes directly after a single bout of exercise, but rather the endurance training effect >24 h after the latest training session.

van Boom et al. (2023) measured CS activity in *post‐mortem* muscle samples from 35 euthanized dogs (aged 1–18 years) across 16 different breeds, including mixed breeds. The study reported CS activity levels between 38 and 80 μmol·min^−1^·g protein^−1^ in the *m*. *triceps brachii* (forelimb) and 33–66 μmol·min^−1^·g protein^−1^ in the *m. vastus lateralis* (hindleg). These values are significantly lower than those found in Siberian huskies (mean values: 372 and 324 μmol·min^−1^·g protein^−1^ off‐season and during racing‐season, respectively) and Alaskan huskies (240 and 537 μmol·min^−1^·g protein^−1^ off season and during racing‐season).

### 4.2 Mitochondrial respiration

In the present study, mitochondrial respiration measurements were performed using previously frozen SM homogenates, in which the integrity of the mitochondrial membranes has been lost. However, by adding exogenous cytochrome C to the homogenate, the mitochondrial electron transport chain (ETS) is re‐constituted so that the freeze‐thawed samples still have intact ETS complexes. This approach has been documented to maintain the mitochondrial ability to consume O_2_ at 90%–95% of the normal maximal respiratory capacity with fresh SM samples (Acin‐Perez et al., 2020).

To account for differences in protein content and CS activity across groups and individuals, we opted to normalize our respiratory data relative to mass, protein, and CS activity. Notably, many of the significant differences observed in respiration normalized to mass or protein were no longer evident when normalized to CS activity. This indicates that most of the training‐induced changes in respiratory capacity were likely driven by alterations in mitochondrial density. However, the data revealed some intriguing and consistent patterns, including a more pronounced increase in CII activity compared to CI activity in raced versus non‐raced dogs of both groups.

#### 4.2.1 Changes in CI and CII capacities‐possible implications

This study is the first to compare the effect of endurance training on mitochondrial respiratory capacity between Siberian and Alaskan huskies. During racing season, mitochondrial complex II (CII) respiration was found to increase in Siberian huskies (+75%) and even more so in Alaskan huskies (+129%) (Table 3). CII catalyzes the oxidation of succinate to fumarate, producing FADH_2_ as an electron donor to coenzyme Q in the ETS (Bezawork‐Geleta et al., 2017; Gnaiger, 2024). Hence, the observed increase in oxygen fluxes in the SM homogenates from the raced huskies following succinate addition suggests enhanced metabolic flux through the TCA cycle and ETS. CII also plays a regulatory role by detecting changes in metabolic demand and activating the TCA cycle to oxidize acetyl‐CoA, a function that may be particularly important during periods of low oxygen availability or heightened energy demand (Gnaiger, 2024). An increase – although lower (+25%) – has previously been reported in CII respiration in raced vs. non‐raced Alaskan huskies (Miller et al., 2017), and humans after high‐intensity interval training (Batterson et al., 2023), while Davis & Barret (2021) found no significant changes in CII respiration in response to training/activity, in their Alaskan huskies (Table 3).

**TABLE 3:** Comparative data on training‐induced changes in CI and CII respiration (mass‐specific) in SM mitochondria.

Animal model (muscle, training)	CI	CII	References
Alaskan huskies			
bf, endurance trained	+36%	+25%	Miller et al. (2017)
bf, endurance trained	+25%	NSC	Davis & Barrett (2021)
bf, endurance trained	+35%	+129%	Current study
Siberian husky			
bf, endurance trained	NSC	+75%	Current study
Horses			
gm, endurance trained	+34%	ND	Votion et al. (2010)
tb, endurance trained	+67%	ND	Votion et al. (2010)
Humans			
vl, high‐intensity interval training	+18%	+24%	Batterson et al. (2023)

Abbreviations: bf, *m. biceps femoris*; gm, *m. gluteus medius*; ND, not determined; NSC, no significant change; tb, *m. triceps brachii*; vl, *m*. *vastus lateralis*.

Raced Alaskan huskies also exhibited a 35% increase in SM mitochondrial complex I (CI) respiration, aligning with data from two previous studies of Iditarod‐raced Alaskan huskies (Davis & Barrett, 2021; Miller et al., 2017) (Table 3). CI respiration is also seen to increase in endurance‐trained horses, although with differences found between muscles (Table 3) (Votion et al., 2010). Increased CI activity (+18%) following training has also been reported in humans (Batterson et al., 2023) (Table 3). NADH, donating electrons to CI, is primarily generated via glycolysis and the TCA cycle. The observed and reported exercise‐linked increase in CI respiration suggests enhanced capacity for carbohydrate utilization, which is a key fuel at high exercise intensities (Van Loon et al., 2001).

Unlike other mammalian models studied, Siberian huskies did not show a significant increase in CI respiration in response to endurance training; they only increased CII mitochondrial respiratory capacity (Table 3). This can indicate a greater reliance on fat utilization, a characteristic that may reflect their ancestral lineage as Arctic sled dogs adapted to utilize fat‐rich diets (Sinding et al., 2020), which can enable sustained lipid‐based energy production during high metabolic demand (Panov et al., 2024). High‐fat diets were also found to enhance cardiac mitochondrial CII activity in mice as compared to normal or low‐fat diets, especially following hypoxic/re‐oxygenation (Zhu et al., 2020). Most commercial racing diets for sled dogs consist of kibble with >16,000 kJ·kg^−1^ (Davis, 2021) to cover the high energy need during long distance races (Hinchcliff et al., 1997; Loftus et al., 2014). These diets contain large amounts of fat (20%–30% on a dry matter basis), high proportions of protein (>30%), and typically 10%–15% carbohydrates, to help maximize caloric intake (Davis, 2021). In addition, raw meat and fish are used to increase palatability and ensure intake of the “kibble stew” (Davis, 2021). Intake of diets rich in fats and proteins also helps preserve muscle glycogen stores during exercise and decrease the likelihood of musculoskeletal injuries in the sled dogs (Reynolds et al., 1995, 1999).

Endurance‐trained Alaskan huskies appear to have greater mitochondrial flexibility than Siberian huskies, utilizing both CI and CII capacity more effectively. This may reflect their combined genetic heredity from ancient Arctic sled dogs and from other dog breeds that are better adapted for starch utilization. Tosi and coworkers (2021) suggested that Alaskan huskies rely more on carbohydrates than fats during multiday submaximal exercise, as supported by increased glucose transporter expression (GLUT1, GLUT4, and GLUT12) in SM of endurance‐trained Alaskan huskies (Barrett & Scott Davis, 2023). Increased SM CII activity in endurance‐trained dogs, as reported in the present study and in the study by Miller et al. (2017), may potentially reflect increased capacity for fatty acid utilization in endurance‐trained dogs, which aligns with prior studies (McKenzie et al., 2005, 2008) showing Alaskan huskies avoid glycogen depletion during prolonged running despite low carbohydrate intake (McKenzie et al., 2005). These adaptations may give them a competitive advantage in long‐distance races. Further research is needed to confirm these hypotheses.

#### 4.2.2 Methodological approach

We have, unlike the cited studies above (Table 3), used previously frozen tissue in which the respiratory complexes are uncoupled from the rest of the cellular metabolism, including the TCA cycle, β‐oxidation, and other metabolism‐modifying mechanisms, such as cellular and intracellular transport of substrates. ETS complexes are known to associate into so‐called supercomplexes (SC), whose formation can be enhanced by both pathological stresses and exercise (Greggio et al., 2017). SCs can be conserved during freezing/thawing processes (Acín‐Pérez et al., 2008), but how the formation of supercomplexes impacts, for example, CII activity is not well studied. Two different subpopulations of coenzyme Q (CoQ), one trapped in SCs for NADH (CI) and another free in the inner membrane for CII and FAD‐dependent enzymes, have been identified (Lapuente‐Brun et al., 2013). Consequently, the observed increase in SM CII activity in this study may indeed reflect enhanced fatty acid utilization.

Previous studies (Davis & Barrett, 2021; Miller et al., 2017) examined muscle biopsies from Alaskan huskies that had participated in the Iditarod (1600 km) and covered a greater total training distance (4500 km), whereas the current study focused on dogs competing in middle‐distance races (50–245 km) with cumulative training distances of 3700 km (Siberian huskies) and 2500 km (Alaskan huskies) during the season of the sampling.

Differences in diets ingested can also impact body and SM metabolism on the day of data sampling. Diets with high fat contents will trigger metabolic responses that facilitate lipid utilization, transport and oxidation more strongly than high carbohydrate diets (Fritzen et al., 2020). The fat content was similar (21%) among the two diets used for the Siberian and the Alaska huskies in this study, but dietary intake and digestibility between the two groups were not measured and may have affected the mitochondrial responses observed. More research is needed to determine which dietary fats best supports performance. And future studies investigating effects of endurance training on mitochondrial function and substrate utilization should also be careful to use similar diets in terms of dietary content when comparing different types of sled dogs.

#### 4.2.3 ROX and the production of ROS

We found a consistent reduction in residual oxygen consumption (ROX) in SM homogenates from raced Siberian and Alaskan huskies when normalized to protein content, which suggests that oxygen consumption for non‐ETS processes is reduced. ROX measurements in previously frozen tissue are not directly comparable to LEAK states in fresh tissue, where leakage of protons back across the inner mitochondrial membrane increases oxygen consumption and reduces ATP production. There are no studies directly comparing frozen and fresh SM tissue in terms of ROX and LEAK values. Although speculative, one cannot exclude that some of the same mechanisms influencing mitochondrial LEAK states can also affect ROX states in pre‐frozen homogenates. Several physiological and pathophysiological stressors, including cold exposure, fatty acids and reactive oxygen species (ROS), may increase LEAK by increased expression or activation of uncoupling proteins. Miller and colleagues (2017) found decreased LEAK in permeabilized SM fibers from raced Alaskan huskies when using fatty acids as substrates, suggesting a lower endogenous uncoupling and higher mitochondrial efficiency. During prolonged, strenuous exercise, SM hyperthermia may develop, which could exert adverse effects on muscle metabolism and limit work performance (Kozlowski et al., 1985). Our data support the idea that reduced ROX and LEAK in SM could be a conditioning response to periods of extreme heat production in raced huskies (Miller et al., 2017), as compared to the off‐season when exposure to cold may activate uncoupling of mitochondria to preserve body heat. Davis and Barrett (2021) found elevated tissue temperatures (from 38°C to 44°C) to increase LEAK in permeabilized SM fibers from Alaskan huskies, both in trained (62%) and in non‐trained dogs (98%). In addition, training alone also induced LEAK in mitochondria respiring on CI substrates. The authors proposed that this increase in LEAK could be a mechanism to reduce ROS production.

Oxygen tension is a key factor regulating ROS production during respirometer analysis in SM preparations (Li Puma et al., 2020). To ensure accurate and comparable results, ROS production should be measured within a consistent and narrow range of chamber O_2_ concentrations across experimental groups during respiratory analyses (Li Puma et al., 2020), as was done in our experiment (Table 2). No differences were found in ROS production between groups during electron flow through CII mediated by succinate. The inhibition of electron flow through addition of rotenone (selective inhibitor of CI) and antimycin A (AntA, inhibits CIII) profoundly increased ROS, in line with previous reports (Quinlan et al., 2012). The observed decrease in oxygen consumption in the ROX state was associated with a large increase in mass‐, protein‐ and flux‐specific ROS production in both raced Siberian and Alaskan husky SM homogenates. This is somehow surprising given that ROX‐respiration was more efficient in the raced animals, and that processes that increase uncoupling may be activated by ROS (Echtay & Brand, 2007). ROS‐release and mitochondrial uncoupling is, however, a sum of many processes in the cell and the mitochondria and the effects of strenuous exercise on these processes have not been studied in frozen SM homogenates previously. Our data on increased ROS release from SM mitochondria following exercise is, however, consistent with previous reports (Sahlin et al., 2010), and redox‐regulation has been shown to be important in the mitochondrial adaptation to exercise (He et al., 2016). Inhibiting ROS production in CIII by ROS‐inhibitor 3 reduced protein‐specific ROS production by 51% in non‐raced and 36% in raced Alaskan huskies, respectively, and by 42% versus 27% in non‐raced and raced Siberian huskies, respectively, but the trends between groups remained suggesting that training‐induced adaptations are made through modification of other ROS‐producing sources.

No previous studies have compared the impact of sex on the SM adaptations to exercise in huskies, but we know from human studies that there are substantial differences in terms of exercise response (Landen et al., 2023). Although our initial design was not made to study sex differences, our data clearly suggest very similar mitochondrial responses to long term endurance training in males and females, with very similar trends in both respiratory function and ROS production.

### 4.3 Refined sampling procedure

This study evaluated an alternative sampling approach (Hawley & Wetmore, 2010) using local anesthesia and a micro biopsy gun to obtain muscle micro‐biopsies from awake dogs. To further minimize procedural strain, dogs received an NSAID 2 h in advance of the procedure. The NSAID was administered for 3 days in total as a precautionary measure, not in response to observed pain or adverse effects. No signs of discomfort were noted in any of the dogs. The sampling environment was also adjusted to fit the individual dogs, reducing the overall stress for the animals.

Micro‐biopsy procedures are a commonly used method to obtain muscle samples from humans, and a preferred approach in studies that require repeated sampling from the same individual (Hayot et al., 2005; Newmire & Willoughby, 2022). Previous studies on SM mitochondrial respiration in Alaskan huskies have used SM biopsies obtained during general, light anesthesia (Davis & Barrett, 2021; Miller et al., 2017). While the biopsy needle used in our study was similar in size to those used in previous research, our protocol avoided sedation entirely, thereby reducing the overall physiological burden on the animals. Our method proved effective in minimizing distress and maintaining animal welfare and may be particularly beneficial for future studies requiring repeated sampling or field‐based protocols.

## 5 LIMITATIONS AND FUTURE DIRECTIONS

While our findings provide valuable insights, the current study was conducted with a relatively small number of dogs from two different kennels, which may not fully represent the broader populations of these breeds. Future studies should standardize dietary intake to isolate the effects of endurance training on mitochondrial function. Training distances and intensities varied between the two groups, as well, with Alaskan huskies logging shorter training distances than the Siberian huskies. Also, responses to acute exercise might differ from the longer‐term responses to systematic endurance exercise training, and one cannot exclude potential effects of acute physical activity as off‐season dogs are free to be active in their kennels. Although exogenous cytochrome C was added to reconstitute the electron transfer system, the results will not fully reflect the in vivo mitochondrial function, which is regulated by a vast number of factors within the SM cells. It should also be noted that there are some indications that local anesthetics can reduce mitochondrial respiration in samples obtained (Lucchinetti et al., 2012).

## 6 CONCLUSIONS

Siberian and Alaskan huskies were shown to attain similar SM mitochondrial respiration capacities and densities through endurance training. Still, despite logging shorter training distances (2700 vs. 3700 km), raced Alaskan huskies exhibited larger increases than Siberian huskies in SM protein content, CS activity, and respiratory capacities, compared to non‐raced levels. Many of the significant differences observed in mass‐ and protein‐adjusted respiration were lost when normalized to CS activity, suggesting that most of the training‐induced increases in respiratory capacity were due to increased mitochondrial density (270% increase in Alaskan huskies and a 124% increase in Siberian huskies). Still, we found a consistent increase in CII activity in both Siberian and Alaskan huskies, and a smaller but still significant increase in protein‐specific CI activity in the Alaskan huskies. The increase in mass‐specific CII respiration during the racing season suggests an enhanced capacity for fat utilization. This metabolic response is crucial for prolonged, moderate‐intensity exercise, where fatty acids serve as a major fuel. The notable increase in mass‐specific CI respiration in Alaskan huskies indicates an increased potential for oxidizing NADH‐generating substrates. In contrast, raced Siberian huskies showed a reduction in protein‐specific CI respiration, suggesting breed‐specific differences in mitochondrial responses to training. Both breeds demonstrated a reduction in protein‐specific ROX during the racing season, indicating more efficient mitochondrial function with less energy wasted on non‐ATP producing activities. This was associated with an increase in the production of ROS when the flow of electrons through the complexes of the ETS was blocked. A minimally invasive sampling approach, including NSAIDs, a light oral sedation, local anesthesia, a micro biopsy gun, and individualized environments to minimize distress secured good animal welfare and provided a practical method for field‐based or repeated SM biopsies without general anesthesia.

## AUTHOR CONTRIBUTIONS

Silje Sælen‐Helgesson, Monica Alterskjær Sundset, Ingebjørg Helena Nymo, Lars P. Folkow, Shona Hiedi Wood, and Chiara Ciccone conceived and designed the research project. Monica Alterskjær Sundset trained and took care of animals together with the mushers. Silje Sælen‐Helgesson, Ingebjørg Helena Nymo, and Monica Alterskjær Sundset sampled the animals together with musher Ida‐Helene Sivertsen. Silje Sælen‐Helgesson and Trine Lund performed the work in the lab. Anne Dragøy Hafstad prepared the figures and analyzed the data. Monica Alterskjær Sundset drafted the manuscript. The graphical abstract figure was prepared by Trine Lund in BioRender https://BioRender.com/j53z4hu. Photos are taken by Monica Alterskjær Sundset. All authors contributed to interpreting the results of experiments, edited, revised, and approved the final version of the manuscript.

## FUNDING INFORMATION

The work was supported by grants from the Tromsø Forskningsstiftelse (TFS) starter grant TFS2016SW and the TFS infrastructure grant (IS3_17_SW) awarded to S.H.W.

## CONFLICT OF INTEREST STATEMENT

We are grateful for the support of Merit Medicals Norway AS who provided highly essential micro‐biopsy equipment for the muscle sampling in this project. Monica Alterskjær Sundset is the owner of Kennel Snykovet and five of the Siberian huskies in this study.

## ETHICS STATEMENT

This study was approved by the Norwegian Food Safety Authority (FOTS ID 28932), complying with the Norwegian and European legislation for use of animals in research. Researchers involved in the sampling were trained and approved for experimental animal science. One of them (Ingebjørg H. Nymo) is also an authorized veterinarian. Importantly, animal caretaker and musher Ida‐Helene Sivertsen participated in the sampling of all the dogs.

## Data Availability

Background data from this study are available in DataverseNO https://doi.org/10.18710/NJW4SQ.

## References

[phy270725-bib-0001] Acin‐Perez, R. , Benador, I. Y. , Petcherski, A. , Veliova, M. , Benavides, G. A. , Lagarrigue, S. , Caudal, A. , Vergnes, L. , Murphy, A. N. , & Karamanlidis, G. (2020). A novel approach to measure mitochondrial respiration in frozen biological samples. The EMBO Journal, 39, e104073. 32432379 10.15252/embj.2019104073 PMC7327496

[phy270725-bib-0002] Acín‐Pérez, R. , Fernández‐Silva, P. , Peleato, M. L. , Pérez‐Martos, A. , & Enriquez, J. A. (2008). Respiratory active mitochondrial supercomplexes. Molecular Cell, 32, 529–539. 19026783 10.1016/j.molcel.2008.10.021

[phy270725-bib-0003] Araki, T. (1977). Freezing injury in mitochondrial membranes. I. Susceptible components in the oxidation systems of frozen and thawed rabbit liver mitochondria. Cryobiology, 14, 144–150. 558865 10.1016/0011-2240(77)90134-1

[phy270725-bib-0004] Barrett, M. R. , & Scott Davis, M. (2023). Conditioning‐induced expression of novel glucose transporters in canine skeletal muscle homogenate. PLoS One, 18, e0285424. 37134107 10.1371/journal.pone.0285424 PMC10155965

[phy270725-bib-0005] Batterson, P. M. , McGowan, E. M. , Stierwalt, H. D. , Ehrlicher, S. E. , Newsom, S. A. , & Robinson, M. M. (2023). Two weeks of high‐intensity interval training increases skeletal muscle mitochondrial respiration via complex‐specific remodeling in sedentary humans. Journal of Applied Physiology (1985), 134, 339–355. 10.1152/japplphysiol.00467.2022 36603044

[phy270725-bib-0006] Bezawork‐Geleta, A. , Rohlena, J. , Dong, L. , Pacak, K. , & Neuzil, J. (2017). Mitochondrial complex II: At the crossroads. Trends in Biochemical Sciences, 42, 312–325. 28185716 10.1016/j.tibs.2017.01.003 PMC7441821

[phy270725-bib-0007] Carpenter, D. H. , & Cooper, R. C. (2000). Mini review of canine stifle joint anatomy. Anatomia, Histologia, Embryologia, 29, 321–329. 11199475 10.1046/j.1439-0264.2000.00289.x

[phy270725-bib-0008] Chinopoulos, C. (2019). Succinate in ischemia: Where does it come from? The International Journal of Biochemistry & Cell Biology, 115, 105580. 31394174 10.1016/j.biocel.2019.105580

[phy270725-bib-0009] Davis, M. S. (2021). Glucocentric metabolism in ultra‐endurance sled dogs. Integrative and Comparative Biology, 61, 103–109. 33871632 10.1093/icb/icab026

[phy270725-bib-0010] Davis, M. S. , & Barrett, M. R. (2021). Effect of conditioning and physiological hyperthermia on canine skeletal muscle mitochondrial oxygen consumption. Journal of Applied Physiology, 130, 1317–1325. 33661725 10.1152/japplphysiol.00969.2020

[phy270725-bib-0011] Doerrier, C. , Garcia‐Souza, L. F. , Krumschnabel, G. , Wohlfarter, Y. , Mészáros, A. T. , & Gnaiger, E. (2018). High‐resolution fluorespirometry and OXPHOS protocols for human cells, permeabilized fibers from small biopsies of muscle, and isolated mitochondria. In Mitochondrial Bioenergetics: Methods and Protocols (pp. 31–70). Springer. 10.1007/978-1-4939-7831-1_3 29850993

[phy270725-bib-0012] Draxl, A. , Eigentler, A. , & Gnaiger, E. (2013). PBI‐shredder HRR‐set: Preparation of tissue homogenates for diagnosis of mitochondrial respiratory function. Mitochondrial Physiol Netw, 17, 1–8.

[phy270725-bib-0013] Ebanks, B. , Kwiecinska, P. , Moisoi, N. , & Chakrabarti, L. (2023). A method to assess the mitochondrial respiratory capacity of complexes I and II from frozen tissue using the Oroboros O2k‐FluoRespirometer. PLoS One, 18, e0276147. 37486925 10.1371/journal.pone.0276147 PMC10365301

[phy270725-bib-0014] Echtay, K. S. , & Brand, M. D. (2007). 4‐hydroxy‐2‐nonenal and uncoupling proteins: An approach for regulation of mitochondrial ROS production. Redox Report, 12, 26–29. 17263904 10.1179/135100007X162158

[phy270725-bib-0015] Egan, B. , O'Connor, P. L. , Zierath, J. R. , & O'Gorman, D. J. (2013). Time course analysis reveals gene‐specific transcript and protein kinetics of adaptation to short‐term aerobic exercise training in human skeletal muscle. PLoS One, 8, e74098. 24069271 10.1371/journal.pone.0074098 PMC3771935

[phy270725-bib-0016] Eigentler, A. , Draxl, A. , Wiethüchter, A. , Kuznetsov, A. , Lassing, B. , & Gnaiger, E. (2015). Laboratory protocol: Citrate synthase a mitochondrial marker enzyme. Mitochondrial Physiology Network, 17, 1–11.

[phy270725-bib-0017] Evans, H. E. , & Lahunta, A. (2013). Miller's Anatomy of the Dog. Elsevier Saunders.

[phy270725-bib-0018] Feuerborn, T. R. , Carmagnini, A. , Losey, R. J. , Nomokonova, T. , Askeyev, A. , Askeyev, I. , Askeyev, O. , Antipina, E. E. , Appelt, M. , & Bachura, O. P. (2021). Modern Siberian dog ancestry was shaped by several thousand years of Eurasian‐wide trade and human dispersal. Proceedings of the National Academy of Sciences, 118, e2100338118. 10.1073/pnas.2100338118 PMC8488619 34544854

[phy270725-bib-0019] Fritzen, A. M. , Lundsgaard, A.‐M. , & Kiens, B. (2020). Tuning fatty acid oxidation in skeletal muscle with dietary fat and exercise. Nature Reviews Endocrinology, 16, 683–696. 10.1038/s41574-020-0405-1 32963340

[phy270725-bib-0020] Gerth, N. , Sum, S. , Jackson, S. , & Starck, J. M. (2009). Muscle plasticity of Inuit sled dogs in Greenland. Journal of Experimental Biology, 212, 1131–1139. 19329747 10.1242/jeb.028324

[phy270725-bib-0021] Gibala, M. J. , Little, J. P. , van Essen, M. , Wilkin, G. P. , Burgomaster, K. A. , Safdar, A. , Raha, S. , & Tarnopolsky, M. A. (2006). Short‐term sprint interval versus traditional endurance training: Similar initial adaptations in human skeletal muscle and exercise performance. The Journal of Physiology, 575, 901–911. 16825308 10.1113/jphysiol.2006.112094 PMC1995688

[phy270725-bib-0022] Gnaiger, E. (2009). Capacity of oxidative phosphorylation in human skeletal muscle: New perspectives of mitochondrial physiology. The International Journal of Biochemistry & Cell Biology, 41, 1837–1845. 19467914 10.1016/j.biocel.2009.03.013

[phy270725-bib-0023] Gnaiger, E. (2024). Complex II ambiguities—FADH2 in the electron transfer system. Journal of Biological Chemistry, 300, 105470. 38118236 10.1016/j.jbc.2023.105470 PMC10772739

[phy270725-bib-0024] Greggio, C. , Jha, P. , Kulkarni, S. S. , Lagarrigue, S. , Broskey, N. T. , Boutant, M. , Wang, X. , Alonso, S. C. , Ofori, E. , & Auwerx, J. (2017). Enhanced respiratory chain supercomplex formation in response to exercise in human skeletal muscle. Cell Metabolism, 25, 301–311. 27916530 10.1016/j.cmet.2016.11.004

[phy270725-bib-0025] Hafstad, A. D. , Boardman, N. T. , Lund, J. , Hagve, M. , Khalid, A. M. , Wisløff, U. , Larsen, T. S. , & Aasum, E. (2011). High intensity interval training alters substrate utilization and reduces oxygen consumption in the heart. Journal of Applied Physiology, 111, 1235–1241. 21836050 10.1152/japplphysiol.00594.2011

[phy270725-bib-0026] Hansen, S. S. , Pedersen, T. M. , Marin, J. , Boardman, N. T. , Shah, A. M. , Aasum, E. , & Hafstad, A. D. (2022). Overexpression of NOX2 exacerbates AngII‐mediated cardiac dysfunction and metabolic remodelling. Antioxidants, 11, 143. 35052647 10.3390/antiox11010143 PMC8772838

[phy270725-bib-0027] Hawley, A. T. , & Wetmore, L. A. (2010). Identification of single nucleotide polymorphisms within exon 1 of the canine mu‐opioid receptor gene. Veterinary Anaesthesia and Analgesia, 37, 79–82. 20017823 10.1111/j.1467-2995.2009.00506.x

[phy270725-bib-0028] Hayot, M. , Michaud, A. , Koechlin, C. , Caron, M. , Leblanc, P. , Prefaut, C. , & Maltais, F. (2005). Skeletal muscle microbiopsy: A validation study of a minimally invasive technique. European Respiratory Journal, 25, 431–440. 15738285 10.1183/09031936.05.00053404

[phy270725-bib-0029] He, F. , Li, J. , Liu, Z. , Chuang, C.‐C. , Yang, W. , & Zuo, L. (2016). Redox mechanism of reactive oxygen species in exercise. Frontiers in Physiology, 7, 486. 27872595 10.3389/fphys.2016.00486 PMC5097959

[phy270725-bib-0030] Hinchcliff, K. W. , Reinhart, G. A. , Burr, J. R. , Schreier, C. J. , & Swenson, R. A. (1997). Metabolizable energy intake and sustained energy expenditure of Alaskan sled dogs during heavy exertion in the cold. American Journal of Veterinary Research, 58, 1457–1462. 9401699

[phy270725-bib-0031] Hood, D. A. , Memme, J. M. , Oliveira, A. N. , & Triolo, M. (2019). Maintenance of skeletal muscle mitochondria in health, exercise, and aging. Annual Review of Physiology, 81, 19–41. 10.1146/annurev-physiol-020518-114310 30216742

[phy270725-bib-0032] Huertas, J. R. , Casuso, R. A. , Agustín, P. H. , & Cogliati, S. (2019). Stay fit, stay young: Mitochondria in movement: The role of exercise in the new mitochondrial paradigm. Oxidative Medicine and Cellular Longevity, 2019, 7058350. 31320983 10.1155/2019/7058350 PMC6607712

[phy270725-bib-0033] Huson, H. J. , Parker, H. G. , Runstadler, J. , & Ostrander, E. A. (2010). A genetic dissection of breed composition and performance enhancement in the Alaskan sled dog. BMC Genetics, 11, 1–14. 20649949 10.1186/1471-2156-11-71 PMC2920855

[phy270725-bib-0034] Huson, H. J. , Srikanth, K. , & Ellis, K. M. (2025). Breeding selection for US Siberian huskies has altered genes regulating metabolism, endurance, development, body conformation, immune function, and behavior. Genes, 16, 1355. 41300807 10.3390/genes16111355 PMC12652727

[phy270725-bib-0035] Korpivaara, M. , Huhtinen, M. , Aspegrén, J. , & Overall, K. (2021). Dexmedetomidine oromucosal gel reduces fear and anxiety in dogs during veterinary visits: A randomised, double‐blind, placebo‐controlled clinical pilot study. Veterinary Record, 189, e832. 34448217 10.1002/vetr.832

[phy270725-bib-0036] Kozlowski, S. , Brzezinska, Z. , Kruk, B. , Kaciuba‐Uscilko, H. , Greenleaf, J. , & Nazar, K. (1985). Exercise hyperthermia as a factor limiting physical performance: Temperature effect on muscle metabolism. Journal of Applied Physiology, 59, 766–773. 4055565 10.1152/jappl.1985.59.3.766

[phy270725-bib-0037] Landen, S. , Hiam, D. , Voisin, S. , Jacques, M. , Lamon, S. , & Eynon, N. (2023). Physiological and molecular sex differences in human skeletal muscle in response to exercise training. The Journal of Physiology, 601, 419–434. 34762308 10.1113/JP279499

[phy270725-bib-0038] Lapuente‐Brun, E. , Moreno‐Loshuertos, R. , Acín‐Pérez, R. , Latorre‐Pellicer, A. , Colás, C. , Balsa, E. , Perales‐Clemente, E. , Quirós, P. M. , Calvo, E. , & Rodríguez‐Hernández, M. (2013). Supercomplex assembly determines electron flux in the mitochondrial electron transport chain. Science, 340, 1567–1570. 23812712 10.1126/science.1230381

[phy270725-bib-0039] Latham, C. M. , Guy, C. P. , Wesolowski, L. T. , & White‐Springer, S. H. (2022). Fueling equine performance: Importance of mitochondrial phenotype in equine athletes. Animal Frontiers, 12, 6–14. 10.1093/af/vfac023 PMC9197311 35711513

[phy270725-bib-0040] Li Puma, L. C. , Hedges, M. , Heckman, J. M. , Mathias, A. B. , Engstrom, M. R. , Brown, A. B. , & Chicco, A. J. (2020). Experimental oxygen concentration influences rates of mitochondrial hydrogen peroxide release from cardiac and skeletal muscle preparations. American Journal of Physiology. Regulatory, Integrative and Comparative Physiology, 318, R972–R980. 32233925 10.1152/ajpregu.00227.2019

[phy270725-bib-0041] Loftus, J. P. , Yazwinski, M. , Milizio, J. G. , & Wakshlag, J. J. (2014). Energy requirements for racing endurance sled dogs. Journal of Nutritional Science, 3, e34. 26101603 10.1017/jns.2014.31 PMC4473159

[phy270725-bib-0042] Lucchinetti, E. , Awad, A. E. , Rahman, M. , Feng, J. , Lou, P.‐H. , Zhang, L. , Ionescu, L. , Lemieux, H. , Thébaud, B. , & Zaugg, M. (2012). Antiproliferative effects of local anesthetics on mesenchymal stem cells: Potential implications for tumor spreading and wound healing. Anesthesiology, 116, 841–856. 22343474 10.1097/ALN.0b013e31824babfe

[phy270725-bib-0043] Makrecka‐Kuka, M. , Krumschnabel, G. , & Gnaiger, E. (2015). High‐resolution respirometry for simultaneous measurement of oxygen and hydrogen peroxide fluxes in permeabilized cells, Tissue Homogenate and Isolated Mitochondria. Biomolecules, 5, 1319–1338. 26131977 10.3390/biom5031319 PMC4598754

[phy270725-bib-0044] McKenzie, E. , Holbrook, T. , Williamson, K. , Royer, C. , Valberg, S. , Hinchcliff, K. , Jose‐Cunilleras, E. , Nelson, S. , Willard, M. , & Davis, M. (2005). Recovery of muscle glycogen concentrations in sled dogs during prolonged exercise. Medicine and Science in Sports and Exercise, 37, 1307–1312. 16118576 10.1249/01.mss.0000175086.41080.01

[phy270725-bib-0045] McKenzie, E. C. , Hinchcliff, K. W. , Valberg, S. J. , Williamson, K. K. , Payton, M. E. , & Davis, M. S. (2008). Assessment of alterations in triglyceride and glycogen concentrations in muscle tissue of Alaskan sled dogs during repetitive prolonged exercise. American Journal of Veterinary Research, 69, 1097–1103. 18672977 10.2460/ajvr.69.8.1097

[phy270725-bib-0046] Meinild Lundby, A. K. , Jacobs, R. A. , Gehrig, S. , de Leur, J. , Hauser, M. , Bonne, T. C. , Flück, D. , Dandanell, S. , Kirk, N. , & Kaech, A. (2018). Exercise training increases skeletal muscle mitochondrial volume density by enlargement of existing mitochondria and not de novo biogenesis. Acta Physiologica, 222, e12905. 10.1111/apha.12905 28580772

[phy270725-bib-0047] Miller, B. , Hamilton, K. , Boushel, R. , Williamson, K. , Laner, V. , Gnaiger, E. , & Davis, M. (2017). Mitochondrial respiration in highly aerobic canines in the non‐raced state and after a 1600‐km sled dog race. PLoS One, 12, e0174874. 28445477 10.1371/journal.pone.0174874 PMC5405936

[phy270725-bib-0048] Newmire, D. E. , & Willoughby, D. S. (2022). The skeletal muscle microbiopsy method in exercise and sports science research: A narrative and methodological review. Scandinavian Journal of Medicine & Science in Sports, 32, 1550–1568. 35904526 10.1111/sms.14215

[phy270725-bib-0049] Oliveira, A. N. , & Hood, D. A. (2019). Exercise is mitochondrial medicine for muscle. Sports Medicine and Health Science, 1, 11–18. 35782464 10.1016/j.smhs.2019.08.008 PMC9219266

[phy270725-bib-0050] Panov, A. V. , Mayorov, V. I. , & Dikalov, S. I. (2024). Role of fatty acids β‐oxidation in the metabolic interactions between organs. International Journal of Molecular Sciences, 25, 12740. 39684455 10.3390/ijms252312740 PMC11641656

[phy270725-bib-0051] Petrella, J. K. , Kim, J. S. , Mayhew, D. L. , Cross, J. M. , & Bamman, M. M. (2008). Potent myofiber hypertrophy during resistance training in humans is associated with satellite cell‐mediated myonuclear addition: A cluster analysis. Journal of Applied Physiology (1985), 104, 1736–1742. 10.1152/japplphysiol.01215.2007 18436694

[phy270725-bib-0052] Picard, M. , Gentil, B. J. , McManus, M. J. , White, K. , St. Louis, K. , Gartside, S. E. , Wallace, D. C. , & Turnbull, D. M. (2013). Acute exercise remodels mitochondrial membrane interactions in mouse skeletal muscle. Journal of Applied Physiology, 115, 1562–1571. 23970537 10.1152/japplphysiol.00819.2013 PMC3841825

[phy270725-bib-0053] Quinlan, C. L. , Orr, A. L. , Perevoshchikova, I. V. , Treberg, J. R. , Ackrell, B. A. , & Brand, M. D. (2012). Mitochondrial complex II can generate reactive oxygen species at high rates in both the forward and reverse reactions. Journal of Biological Chemistry, 287, 27255–27264. 22689576 10.1074/jbc.M112.374629 PMC3411067

[phy270725-bib-0054] Reynolds, A. J. , Fuhrer, L. , Dunlap, H. L. , Finke, M. , & Kallfelz, F. A. (1995). Effect of diet and training on muscle glycogen storage and utilization in sled dogs. Journal of Applied Physiology (1985), 79(5), 1601–1607. 10.1152/jappl.1995.79.5.1601 8594020

[phy270725-bib-0055] Reynolds, A. J. , Reinhart, G. A. , Carey, D. P. , Simmerman, D. A. , Frank, D. A. , & Kallfelz, F. A. (1999). Effect of protein intake during training on biochemical and performance variables in sled dogs. American Journal of Veterinary Research, 60, 789–795. 10407468

[phy270725-bib-0056] Sahlin, K. , Shabalina, I. G. , Mattsson, C. M. , Bakkman, L. , Fernström, M. , Rozhdestvenskaya, Z. , Enqvist, J. K. , Nedergaard, J. , Ekblom, B. , & Tonkonogi, M. (2010). Ultraendurance exercise increases the production of reactive oxygen species in isolated mitochondria from human skeletal muscle. Journal of Applied Physiology, 108, 780–787. 20110545 10.1152/japplphysiol.00966.2009 PMC2853199

[phy270725-bib-0057] Sinding, M.‐H. S. , Gopalakrishnan, S. , Ramos‐Madrigal, J. , de Manuel, M. , Pitulko, V. V. , Kuderna, L. , Feuerborn, T. R. , Frantz, L. A. , Vieira, F. G. , & Niemann, J. (2020). Arctic‐adapted dogs emerged at the Pleistocene–Holocene transition. Science, 368, 1495–1499. 32587022 10.1126/science.aaz8599 PMC7116267

[phy270725-bib-0058] Siu, P. M. , Donley, D. A. , Bryner, R. W. , & Alway, S. E. (2003). Citrate synthase expression and enzyme activity after endurance training in cardiac and skeletal muscles. Journal of Applied Physiology, 94, 555–560. 12531911 10.1152/japplphysiol.00821.2002

[phy270725-bib-0059] Smith, T. A. , Srikanth, K. , & Huson, H. J. (2024). Comparative population genomics of Arctic sled dogs reveals a deep and complex history. Genome Biology and Evolution, 16, evae190. 39193769 10.1093/gbe/evae190 PMC11403282

[phy270725-bib-0060] Sports IFoS . (2024). International federation of sleddog sports race rules 2024–2026.

[phy270725-bib-0061] Thorsrud, J. A. , & Huson, H. J. (2021). Description of breed ancestry and genetic health traits in arctic sled dog breeds. Canine Medicine and Genetics, 8, 1–13. 34544496 10.1186/s40575-021-00108-z PMC8454093

[phy270725-bib-0062] Tosi, I. , Art, T. , Boemer, F. , Votion, D.‐M. , & Davis, M. S. (2021). Acylcarnitine profile in Alaskan sled dogs during submaximal multiday exercise points out metabolic flexibility and liver role in energy metabolism. PLoS One, 16, e0256009. 34383825 10.1371/journal.pone.0256009 PMC8360531

[phy270725-bib-0063] van Boom, K. M. , Schoeman, J. P. , Steyl, J. C. , & Kohn, T. A. (2023). Fiber type and metabolic characteristics of skeletal muscle in 16 breeds of domestic dogs. The Anatomical Record, 306, 2572–2586. 36932662 10.1002/ar.25207

[phy270725-bib-0064] Van Loon, L. J. , Greenhaff, P. L. , Constantin‐Teodosiu, D. , Saris, W. H. , & Wagenmakers, A. J. (2001). The effects of increasing exercise intensity on muscle fuel utilisation in humans. The Journal of Physiology, 536, 295–304. 11579177 10.1111/j.1469-7793.2001.00295.x PMC2278845

[phy270725-bib-0065] Vigelsø, A. , Andersen, N. B. , & Dela, F. (2014). The relationship between skeletal muscle mitochondrial citrate synthase activity and whole body oxygen uptake adaptations in response to exercise training. International Journal of Physiology, Pathophysiology and Pharmacology, 6, 84–101. 25057335 PMC4106645

[phy270725-bib-0066] Votion, D. M. , Fraipont, A. , Goachet, A.‐G. , Robert, C. , Van Erck, E. , Amory, H. , Ceusters, J. , de la Rebière Pouya, G. , Franck, T. , & Mouithys‐Mickalad, A. (2010). Alterations in mitochondrial respiratory function in response to endurance training and endurance racing. Equine Veterinary Journal, 42, 268–274. 10.1111/j.2042-3306.2010.00271.x 21059017

[phy270725-bib-0067] Wiegand, G. , & Remington, S. J. (1986). Citrate synthase: Structure, control, and mechanism. Annual Review of Biophysics and Biophysical Chemistry, 15, 97–117. 10.1146/annurev.bb.15.060186.000525 3013232

[phy270725-bib-0068] Xu, Y. , Xue, D. , Bankhead, I. I. I. A. , & Neamati, N. (2020). Why all the fuss about oxidative phosphorylation (OXPHOS)? Journal of Medicinal Chemistry, 63, 14276–14307. 33103432 10.1021/acs.jmedchem.0c01013 PMC9298160

[phy270725-bib-0069] Zhu, S. C. , Chen, C. , Wu, Y. N. , Ahmed, M. , Kitmitto, A. , Greenstein, A. S. , Kim, S. J. , Shao, Y. F. , & Zhang, Y. H. (2020). Cardiac complex II activity is enhanced by fat and mediates greater mitochondrial oxygen consumption following hypoxic re‐oxygenation. Pflügers Archiv/European Journal of Physiology, 472, 367–374. 32078030 10.1007/s00424-020-02355-8

